# Spectral-Coding-Based Compressive Single-Pixel NIR Spectroscopy in the Sub-Millisecond Regime

**DOI:** 10.3390/s21165563

**Published:** 2021-08-18

**Authors:** Paul Gattinger, Ivan Zorin, Christian Rankl, Markus Brandstetter

**Affiliations:** RECENDT—Research Center for Non-Destructive Testing GmbH, Science Park 2, Altenberger Str. 69, 4040 Linz, Austria; paul.gattinger@recendt.at (P.G.); ivan.zorin@recendt.at (I.Z.); christian.rankl@recendt.at (C.R.)

**Keywords:** single-pixel, spectroscopy, near-infrared, DMD, multiplexing, spectral coding, sub-millisecond, compressive measurement

## Abstract

In this contribution, we present a high-speed, multiplex, grating spectrometer based on a spectral coding approach that is founded on principles of compressive sensing. The spectrometer employs a single-pixel InGaAs detector to measure the signals encoded by an amplitude spatial light modulator (digital micromirror device, DMD). This approach leads to a speed advantage and multiplex sensitivity advantage atypical for standard dispersive systems. Exploiting the 18.2 kHz pattern rate of the DMD, we demonstrated 4.2 ms acquisition times for full spectra with a bandwidth of 450 nm (5250–4300 cm^−1^; 1.9–2.33 µm). Due to the programmability of the DMD, spectral regions of interest can be chosen freely, thus reducing acquisition times further, down to the sub-millisecond regime. The adjustable resolving power of the system accessed by means of computer simulations is discussed, quantified for different measurement modes, and verified by comparison with a state-of-the-art Fourier-transform infrared spectrometer. We show measurements of characteristic polymer absorption bands in different operation regimes of the spectrometer. The theoretical multiplex advantage of 8 was experimentally verified by comparison of the noise behavior of the spectral coding approach and a standard line-scan approach.

## 1. Introduction

Infrared (IR) spectroscopy is a well-established analytical method for non-destructive chemical characterization of materials in all aggregate states. Since its beginnings, the field of IR spectroscopy has been dominated by instruments based on thermal emitters. Such instrumentation naturally faces limitations imposed by the low brightness of thermal sources. During the last two decades new measurement technologies have been developed to overcome these limitations, facilitated by novel laser technology, such as quantum cascade lasers [1,2] and supercontinuum laser sources [3,4]. However, these laser sources are rather expensive, which limits their range of application and triggers the development of alternative speed- and sensitivity-enhanced measurement approaches.

Considering the developments in non-laser spectroscopic instrumentation, three conceptual categories of devices can be distinguished: dispersive systems, filter spectrometers (tunable or discrete filters), and the gold-standard technique of Fourier transform spectrometry [5]. As initially developed, dispersive spectrometers dominated in molecular spectroscopy, being capable of providing high acquisition rates with sufficiently high spectral resolution and a relatively simple design [6,7]. State-of-the-art filter-based systems, such as tunable MEMS-based Fabry–Pérot etalons, represent a cost-efficient alternative. However, due to the trade-off between scan speed and spectral resolution, settling times of such filters are usually in the range of tens of milliseconds [8]. In contrast, Fourier transform IR (FTIR) based spectrometers offer inherently high resolution (reciprocal to the scanning range in interferometric systems), broadband operation (limited by the specific detectors responsivity), and transition of the key spectrum reconstruction procedure to the digital domain [9]. The methods of Fourier-transform spectrometry comprise primarily low-coherence interferometric FTIR instruments [10]. Commonly, the approach of FTIR spectrometry holds two inherent advantages over tunable and dispersive systems, namely multiplexing and optical throughput advantages, also known as Fellgett’s and Jacquinot’s advantages, respectively [9,11,12]. The optical throughput—being a function of system geometry—is subject to the optical design (severely limited for slit-based systems), while multiplexing is a property of the detection approach. The multiplex advantage implies that all spectral frequencies of the light source are sampled simultaneously, thus increasing the signal-to-noise ratio (SNR) immensely. Another commonly cited advantage of FTIRs is wavelength accuracy, which is, however, not inherent to FTIR but enabled by the implementation of a reference laser.

In conventional FTIR spectrometers, the autocorrelation function (correlation between the field with its delayed replica, connected with the power spectral density via the Fourier transform) is measured sequentially in time to retrieve IR spectra. Therefore, an intrinsic feature of these instruments is that the measurement time is proportional to the spectral resolution (i.e., the displacement of the scanning mirror; mirror speed can vary for different instruments).

In classical dispersive systems, optical elements such as gratings or prisms are employed to perform the Fourier transform, therefore, eliminating the trade-off between the spectral performance and acquisition speed. This leads to the possibility of fast measurements of spectral regions of interest (SROI), since the spectral resolution is decoupled from the measurement time if array detectors are used. However, since these systems measure only narrow spectral lines per sensing element (spatially or time separated), the multiplex advantage is lost, which causes measured signals to be weak [13]. Therefore, highly sensitive detectors must be used.

The acquisition speed advantage and multiplexing advantage are inherent either for dispersive or for FTIR spectrometers, respectively, showing that these properties are contradictory. In this work, we address this point by demonstrating a dispersive spectrometer that displays both the multiplex advantage, usually nonspecific for such systems, and the speed advantage by using a spectral coding approach based on the principles of compressive sensing (CS).

The emerging field of CS exploits the fact that a sparse signal can be recovered from far fewer samples than those required by the Nyquist–Shannon–Kotelnikov theorem [14,15]. Applications of CS techniques have evolved over the past decade. Besides the employment in the standard vis-imaging scenarios [16,17] and new approaches in combination with deep learning [18], CS has also occupied various specific sectors of applied science such as communication systems, biomedical applications, micro- and nano-electronics, and sound and speech processing [19,20]. A recent article about the application of CS in NIR hyperspectral imaging [21] demonstrated potential advantages over well-established measurement approaches and techniques. For instance, in resonator-based spectroscopy the effective bandwidth can be noticeably broadened by applying CS schemes [22]. Considering the problem raised, the spectral coding approach—based on amplitude light modulation—can be used in dispersive element-based spectrometer designs to attain the multiplex advantage [23]; thus, a single detector can be employed to detect several spectral components simultaneously while the spectrum is being computed for distinctive spatial patterns. We applied a compressive measurement approach similar to CS that allows undersampling of spectra to increase acquisition speeds, i.e., less spatial patterns have to be applied to reconstruct encoded spectral information. The main difference from CS is that smoothness of the spectra is used as a regularization instead of the usual exploitation of signal sparsity. Spatial amplitude modulation can be achieved by using fully integrated spatial light modulators. Following this approach, classical moving parts for scanning, such as rotating gratings or mirrors, become unnecessary and highly sensitive single element detectors can be used as opposed to linear arrays. Experimental realization was reported both for Raman and infrared spectroscopy [24,25,26,27]. This measurement approach enhances sensitivity and speed, which makes applications in the NIR and MIR regime attractive.

In this contribution, we demonstrate an approach that combines the advantages of FTIR instruments with those of dispersive element-based instruments in the NIR spectral region. The developed system is based on a ruled diffraction grating and utilizes spectral coding to achieve spectral multiplexing by means of a MEMS-based digital micromirror device (DMD) [28]. Such an approach has already been realized in commercial NIR systems based on a concept reported in [29]. A performance comparison of this concept to other state-of-the-art NIR spectrometer technologies is shown in [27]. In the present paper we used a laboratory setup with a similar optical approach, however, we focused on harnessing the full potential of the general concept of using spatial light modulators. The programmable DMD makes the systems’ parameters flexible and enables measurements of either the whole spectral band or a dedicated SROI, thus enabling acquisition rates in the millisecond regime while maintaining high spectral resolution. The flexibility of the DMD also makes the implementation of compressive measurements possible, thus, allowing sampling of the whole spectral band or SROI at high resolution with only a fraction of the measurements, resulting in a significant reduction of the acquisition time under the condition that the spectra are smooth.

In the implementation section, the design of the developed system is demonstrated, and its resolution performances and features are investigated by simulations and discussed in detail. In the experimental part, we demonstrate the performance characteristics of our approach in different operational modes (scanning grating spectrometer, spectral coding, and spectral coding combined with a compressive sensing approach). This is then compared to a standard FTIR-spectrometer serving as a gold standard. The theoretical multiplex advantage resulting from the spectral coding principle is experimentally demonstrated by a quantitative analysis of the spectral noise behavior.

## 2. Materials and Methods

### 2.1. Setup

**Figure 1:**
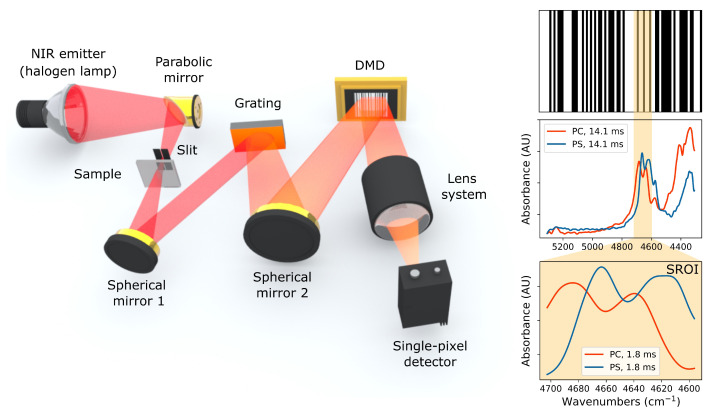
Schematic optical arrangement and principles of operation of the developed dispersive multiplex spectrometer. Thermal emitter radiation (halogen lamp) passes the slit, which determines the optical throughput and the resolution of the system (i.e., spatial size of a single spectral element in the DMD plane); spherical mirror 1 collimates the light and redirects it to the diffraction grating, spherical mirror 2 focuses the spectral components onto the DMD producing an image of the spectrum; the modulated radiation is collected by the lens system and focused onto a single point detector. The inset plots indicate the absorbance spectra (AU, absorbance units) of polystyrene (PS) and polycarbonate (PC) that are encoded by barcode-like modulation patterns. SROI can be obtained by using only a fraction of the modulation bars.

The optical arrangement, depicted in Figure 1, was designed to perform spectroscopic measurements in transmission geometry. Light coming from a thermal emitter (halogen light source, 35 W) is focused via a parabolic mirror (f = 50 mm) onto a slit and then goes through the sample. A standard spherical gold mirror with f = 150 mm collimates the light passing through the slit and reflects it towards the diffraction grating (Thorlabs, Newton, NJ, USA, GR2550-30035, 300 lines/mm) that separates the spectral components. A second standard spherical gold mirror (f = 100 mm) focuses the spectral components onto the DMD (Texas Instruments, Dallas, TX, USA, DLP7000 0.7 XGA 2xLVDS with customized CaF_2_ sealing window), producing a polychromatic image of the entrance slit in the spectral coding plane. The DMD operates in a binary mode by deflecting the mirrors by either −12° or 12° from the flat state. A focusing lens system consisting of a plano-convex CaF_2_ lens (f = 60 mm) and a ZnSe microscope objective (f = 6 mm) is used to collect the spatially modulated radiation coming from the DMD and to focus it on the extended InGaAs chip of the single-pixel detector (Thorlabs, PDA10DT). The synchronization of the system is coordinated by a computer program (Python/Qt based). The software controls the DMD board (Vialux, Chemnitz, Germany, V7001, 18.2 kHz pattern switching rate) that sends master trigger signals when switching patterns, which are used to initiate the sampling cycles of the DAQ-card (Spectrum GmbH, München, Germany, M3i.4142-Exp). Integration of the samples and reconstruction of the spectra by either solving the linear equations (spectral coding) or using optimization algorithms (compressive approach) as well as zero-padding and smoothing (Blackman–Harris) is carried out by the software in post-processing.

### 2.2. Spectrometer Design

**Figure 2:**
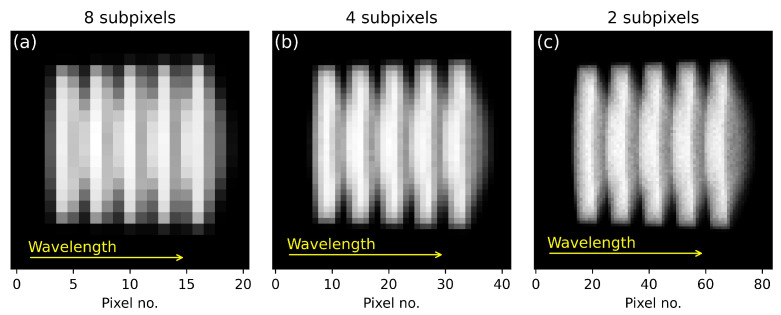
Zemax simulations of linear dispersion in the DMD plane; in each of the illustrations we show five images, corresponding to five different wavelengths of the entrance slit. In the figures, spectrally separated images of the entrance slit with a width of 350 µm are shown. Five wavelength elements with 10.5 nm spectral distance (2180 nm, 2190.5 nm, 2201 nm, 2211.5 nm, 2222 nm) are simulated. For each wavelength element an image of the slit is projected onto the DMD surface; resolution of the slit images for different DMD sub-pixel groupings: (**a**) 128 pixels; 8 sub-pixels per DMD super-pixel, (**b**) 256 pixels; 4 subpixels per DMD super-pixel (**c**) 512 pixels; 2 sub-pixels per DMD super-pixel.

The grating spectrometer was designed to operate in a spectral range between 5250 cm^−1^ and 4300 cm^−1^, a spectral window where many molecules, e.g., a variety of polymers, feature overtone and combination absorption bands. The spectral bandwidth is defined by the angular dispersion of the grating (blazed, 300 lines/mm), the collimation and focusing optics, and the width of the DMD (14 mm). The spectral range and the central wavelength can be adapted flexibly according to the desired spectral range to be covered. The first conceptual part of the setup represents a typical polychromator design where the linear array is replaced by a system consisting of DMD, lens system, and a point detector. The theoretical resolving power of the system is principally limited by the properties of the grating and the central wavelength λ to be resolved. The optical system, in which the grating is embedded, further limits the possible resolution due to inherent aberrations and finite slit width. The latter is in a specific trade-off with the transmitted power. The most pronounced effect reducing the resolution is coma, generated by the high angles and curvatures induced by the compact design (20 × 20 × 5 cm^3^). Astigmatism, which is introduced by using spherical mirrors instead of toroidal shaped optics, does not influence the quality of the spectra since the signal is integrated over the DMD y-axis, orthogonal to the focused spectral component plane. Aside from aforementioned effects of aberrations, for the linear dispersion given by optics, the resolution is restricted by the width of the entrance slit of the system. An entrance slit with 350 µm in width provides enough optical power for fast measurements. The collimating mirror, with a focal distance of 15 cm, and the imaging mirror, with f = 10 cm, lead to a magnification of 0.67. Therefore, the projection image of the entrance slit for a single wavelength component covers a width of 233 µm on the DMD surface. For the chosen spectral bandwidth of 450 nm and a DMD width of 14 mm, this results in a possible resolution of around 7.5 nm. However, in contrast to conventional polychromators, the widely adjustable pixel size (grouping of single pixels to super-pixels) of the DMD plays the third role in the resolution game. In order to investigate the spectral resolution as a function of number of single pixels grouped together in order to form super-pixels and to estimate the effects of aberrations, simulations using an optical design software (Zemax) were performed. Polychromatic imaging of the slit in the DMD plane was simulated. For the simulation, five different wavelengths with a spectral spacing of 10.5 nm starting with 2180 nm were used. In Figure 2 the five projection images of the entrance slit at the DMD, corresponding to the five bars, are shown. Additionally, Figure 2 shows coma, which originates from the big angles occurring in the system (see Figure 1). A single mirror element on the DMD has a width of 13.68 µm. A bar, corresponding to the projection of the entrance slit, has a width of 233 µm. It is evident that in this case, using single DMD mirrors for the spectral coding will oversample the spectral resolution limit and, therefore, is just time consuming. In a tutorial on how to design a spectrometer from Scheeline [13] it is noted that three measurement points within the resolution limit are sufficient for sampling. In order to fulfill this, it is necessary to group 5.7 mirrors together for a super-pixel. The number of total DMD mirrors along the wavelength-axis is 1024. Additionally, we want to use the entire DMD window. Six is not a factor of 1024, therefore, it is convenient to either group 4 or 8 mirror elements to one super-pixel. During the measurement, the single-pixel detector measures the intensity that comes from the “on” mirrors. Four mirror elements conserve the 7.5 nm spectral resolution. Grouping eight mirrors results in worse spectral resolution but provides shorter acquisition times. Eight mirror elements have a width of 109 µm. In the case when the central wavelength lies between two super-pixels, i.e., two neighboring super-pixels along the wavelength axis are evenly illuminated, the width of at least three super-pixels defines the spectral resolution. Therefore, grouping 8 DMD mirror elements to one super-pixel gives a spectral resolution of 10.5 nm. A spectral resolution of 10.5 nm is still sufficient to discriminate certain polymers.

In order to demonstrate the capabilities of the dispersive multiplex system, 1 mm thick films of the two well-known and widely used polymers polycarbonate (PC) and polystyrene (PS) were chosen as suitable specimens. For spectral verification and comparison purposes, measurements with a standard FTIR spectrometer (Bruker Vertex 70, halogen light-source, resolution 12 cm^−1^, 20 kHz mirror frequency, InGaAs detector, CaF_2_ beamsplitter) were performed. The calibration of the spectrometer was carried out via measurements of a PC sample that displays distinctive absorption bands in the spectral region for which the spectrometer was designed. Two absorption bands (at 2064 nm and 2185 nm, R-OH combination bands) were used to calibrate the wavelength axis, since the spectrum is equidistant in wavelengths.

### 2.3. Sensitivity Advantages

Considering the sensitivity advantages in spectrometers, initially introduced and realized with the emergence of FTIR systems, one has to distinguish between two types: Fellgett’s (multiplex) advantage and Jacquinot’s throughput advantage. FTIR spectrometers usually show both of these advantages, as they have big apertures (Jacquinot’s advantage) and sample all wavelengths simultaneously with a single-pixel detector (Fellgett’s advantage). Conventional grating spectrometers commonly have none of these advantages. They have entrance slits, which reduce the throughput and only small fractions at a time of the emitted light are detected by a detector element. In the proposed system, 50% of the spectrometer’s total bandwidth (450 nm) is multiplexed due to the spectral coding approach (50% of the DMD pixels are in the “on” position at the same time). Therefore, the overall intensity is on average 50% higher than that of conventional grating-based spectrometers.

The main advantage compared to FTIR spectrometers is the possibility to measure a SROI (inset in Figure 1), enabled by the programmable flexibility of the DMD. In FTIR spectrometers the measurement time (defined by the scanning ranges and speed) determines the resolution, while the whole spectral band is recorded. In the case of the spectral coding approach (introduced and discussed in the next section) implemented in the developed grating spectrometer, spectral sub-bands or SROI can be sampled, providing the same or even higher spectral resolution and sacrificing only parts of Fellgett’s advantage, depending on how small the SROI is. For instance, if the SROI lies in one half of the DMD width, then only 25% of the spectral components are multiplexed; in the limiting case, narrowing the SROI to a single spectral element will be equivalent to a monochromator line-scan configuration with no sensitivity advantage.

### 2.4. Spectral Coding and Compressive Measurement Approach

The spectral coding approach can be used to realize a grating spectrometer without the application of linear detection arrays and without mechanically moving optics or gratings [29]. The idea is to apply a series of wavelength-dependent modulation masks, measure the encoded signals, and reconstruct the spectrum from those measurements. For this, we used a DMD to turn on and off spectral components. In every modulation, 50% of the spectral components were multiplexed onto a single element detector. Thereby, pseudo-random binary barcode-like patterns were displayed on the DMD, where each pattern was followed by its inverse representation, so that differences in the corresponding signals were measured. Spectra were reconstructed subsequently by solving the resulting system of linear equations

**y = Rx** (1)

where **y** is an m-element vector containing the difference measurements and **R** is a m × n matrix where the individual rows consist of 1 and −1, indicating that the spectral component is turned on at first and turned off subsequently or turned off at first and turned on subsequently, respectively. **x** is an n-element vector representing the original spectrum. If m ≥ n then **x** can be reconstructed using regular least-square optimization, otherwise we have an under-determined linear equation system. Nevertheless, the spectra can be reconstructed using

(2) $$\min_{\hat{x}}{||R\hat{x}-y||}_{2}^{2}+\alpha {\left||\frac{d^{2}\hat{x}}{d\lambda^{2}}|\right|}_{2}^{2}.$$

The first term is a usual data fitting term. The second term is a regularization term that minimizes the second derivative of $\hat{x}$, with α being the regularization parameter. This algorithm finds the approximation $\hat{x}$ to the real spectrum **x** for minimal values of the second derivative along the wavelength axis, with ${||.||}_{2}^{2}$ representing the square of the L2 norm, assuming the measured spectra are smooth. This smoothness assumption is valid due to the limited spectral resolution of the system. Similar to CS, this approach allows for under-sampling by considering a priori knowledge (i.e., smoothness) about the signal. It differs from classical CS where a signal can be reconstructed under the assumption that there exists a sparse representation [30].

## 3. Results

**Figure 3:**
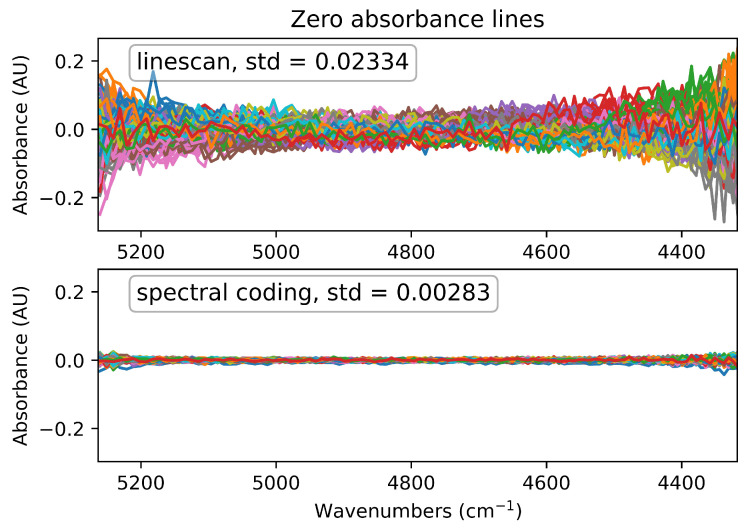
Quantification of the gained multiplex advantage: zero absorbance lines measured using the monochromator mode (line-scan) and the spectral coding mode respectively (125 zero-absorbance lines for both instances); the mean values of the standard deviation (std) of the 100% lines are 0.02334 AU (absorbance units) for the line-scans and 0.00283 AU for the multiplexed measurements, resulting in a factor of 8.2 sensitivity advantage.

In order to demonstrate the multiplex sensitivity advantage over a standard mono- or polychromator configuration, zero-absorbance spectra for two modalities of the system (line-scan and spectral coding) were recorded and analyzed. This method is especially suitable for quantification of spectral noise, as shown in Figure 3, for the line-scan and the spectral coding modality, respectively. It is important to notice the increase of spectral noise towards the edges of the spectral band. This effect occurs due to the geometric mismatch of the DMD and the detector apertures. While the DMD has a rectangular geometry, the detector chip is of circular shape. As further shown in Figure 3, the mean of the standard deviation (std) of 125 zero absorbance lines, which are calculated from the logarithm of the quotient of two blank spectra (no specimen in the beam path), is bigger than for the line-scan mode by a factor 8.2. The multiplex advantage is quantified by the value *N*^1/2^ [11,23], with *N* being the number of DMD super-pixels. In this special case of Hadamard multiplexing the multiplex advantage can be calculated by computing [31,32]

(3) $${\left(\frac{\varepsilon_{\mathrm{line}-\mathrm{scan}}}{\varepsilon_{\mathrm{spectral}\;\mathrm{coding}}}\right)}^{1/2}={\left(\frac{N\sigma^{2}}{Tr\left({\left(H^{-1}\right)}^{T}H^{-1}\right)\sigma^{2}\;}\right)}^{1/2}=N^{1/2}$$

with *ε* denoting the mean square error of a measurement. *σ*^2^ is the rms noise of a single measurement and **H** stands for a Hadamard matrix of dimension *N* × *N*. In the ideal case, where the 1 and −1 entries of the Hadamard matrix could be measured simultaneously with the same detector, this would result in a multiplex advantage of √128. Due to the fact that we have to split the Hadamard matrix into two matrices and therefore have to perform twice as many measurements, the multiplex advantage should be evaluated for twice as many line scans. This means that the result of Equation (3) has to be multiplied by a factor √(1/2). This gives a theoretical multiplex advantage of 8, which is close to the experimentally found multiplex advantage of 8.2, which was evaluated by computing the standard deviation of 125 zero absorbance lines, calculated from 250 spectral coding measurements and 500 line-scan measurements.

**Figure 4:**
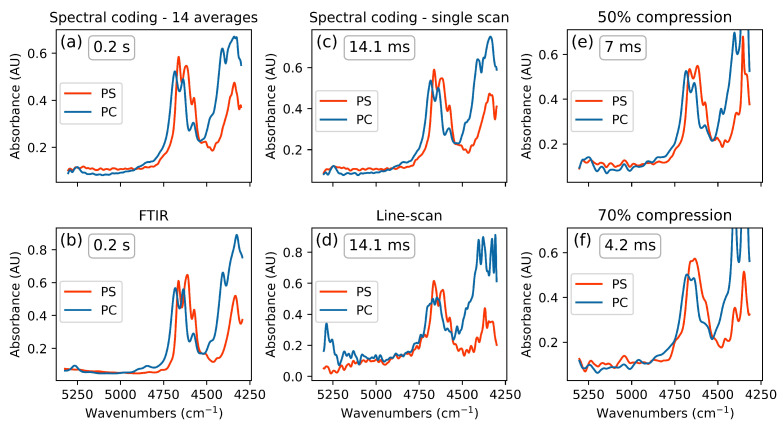
PC and PS absorbance spectra (AU, absorbance units) recorded with different approaches and different resulting acquisition times; (**a**) recorded with the spectral coding-based grating spectrometer; (**b**) reference absorbance spectra recorded with a standard FTIR spectrometer; (**c**) spectral coding approach with a DMD pattern switching rate of 18.2 kHz; (**d**) imitation of a conventional grating spectrometer by making line-scans of the spectra using single bar illumination masks; (**e**) compressive measurement approach with a compression of 50%, a loss in spectral resolution can be observed; (**f**) 70% compression of the measurement leads to a loss in spectral resolution but decreases the acquisition time to below 5 ms.

The measurement time for a single spectrum is mainly defined by the illumination time of each modulation pattern and the number of patterns that are used in total. The measurements shown in Figure 4, except (b), were performed with an illumination time of 55 µs, leading to 14.1 ms pure measurement time per single spectrum when using the spectral coding approach (i.e., 100% of the modulation masks). When introducing compression, the measurement time is given by multiplication of the total measurement time with the fraction of the full set of patterns that is used to sample the spectrum. Thereby, the system becomes even faster at the trade-off of losing spectral resolution. Figure 4a shows the reconstructed spectra recorded using the spectral coding approach. An effective pattern repetition time of 55 µs was used and 14 spectra were averaged in total, yielding an overall measurement time of 0.2 s. When compared to the verification measurements (Figure 4b), it is clearly visible that the spectral resolution of the developed spectral coding spectrometer is comparable to that of the commercial FTIR standard spectrometer, which was set to be 12 cm^−1^. The measurement time of the FTIR spectrometer for a single scan at the given resolution is 0.2 s. This result is in a good agreement with those of the optical design and Zemax simulations, which predicted a spectral resolution below 10.5 nm. The two central bands that are 20 nm apart could be resolved as precisely as with the standard instrument, thus suggesting an approximate spectral resolution of 12 cm^−1^, which is about 5.5 nm in the given spectral range.

All the spectra recorded using the spectral coding approach that are depicted in Figure 4 were zero-filled and Blackman–Harris-filtered. Figure 4d indicates the results when a monochromator approach is used (no multiplexing, equivalent to standard dispersive spectrometers), with an acquisition time for two spectra of 14.1 ms. This measurement was realized by imitating line scans by applying only a single bar pattern per illumination mask on the DMD. In Figure 4e only 50% of the barcode pattern set was used to sample the spectrum, resulting in 50% compression at an acquisition time of 7 ms. Compressing the measurement by 70% reduces the acquisition time to 4.2 ms (Figure 4f) but at the same time a severe trade-off to spectral resolution is made.

**Figure 5:**
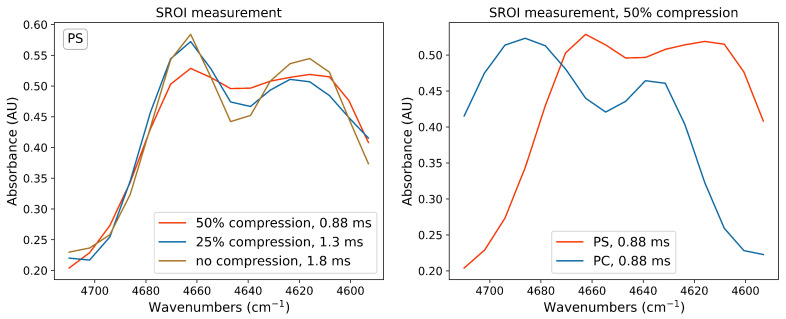
**Left**: SROI measurement with millisecond and sub-millisecond acquisition time and different compressions (raw data, no smoothing or interpolation applied). Only 16 super-pixels per illumination mask were used to sample this SROI, but the spectral bands can still be separated up to compression rates of 50%, where only 8 spatial patterns were used to sample the spectrum; **right**: comparison of 50% compressed absorbance spectra of PS and PC (raw data, no smoothing or interpolation applied).

The high flexibility of the spectral coding spectrometer and the associated speed advantage can be demonstrated by measurements of SROI. For sampling the spectra shown in Figure 5, modulation patterns of 16 super-pixels were used, as indicated in the inset in Figure 1, thus, decreasing the measurement time further, by a factor of 1/8. This results in single spectra acquisition of 1.8 ms. Since the size of the super-pixels remained unchanged, the spectral resolution in the ROI is the same as that for a full 128 pixel measurement. On the left side in Figure 5, the raw, unprocessed 16-point absorbance spectra of PS are shown for different compressions. On the right side we show that the absorption bands of PC and PS can still be resolved when only 50% of the full set of modulation patterns are used to sample the SROI (50% compression), resulting in an acquisition time of 0.88 ms. As mentioned above, the multiplex advantage is extenuated due to the fact that spectral power of only a small sub-band is modulated and measured by the detector.

## 4. Discussion

In this work we introduced a fast and cost-effective spectral coding-based grating spectrometer for the near-infrared spectral domain (5250–4300 cm^−1^; 1.9–2.33 µm). The spectrometer combines desirable advantages of dispersive element-based spectrometers with that of FTIR spectrometers. Spectral flexibility of the system is enabled by use of a programmable spectral light modulator, i.e., a DMD, allowing for spectral coding. With the chosen approach the multiplex advantage can be exploited due to the fact that 50% of the spectral components projected onto the DMD are captured by the detector per measurement, thus providing an increase in sensitivity compared to that of classical dispersive approaches. The spectral resolution of the system was characterized using an optical design software (Zemax), suggesting a spectral resolution better than 10.5 nm for the used number of super-pixels. The implementation of the DMD-based detection allowed for single measurements at a rate of 18.2 kHz, thus enabling sampling times of 14.1 ms for full spectra with 128 pixels. Additionally, we introduced a compressive measurement approach that enabled full spectral acquisition in 4.2 ms. Single SROI of 50 nm bandwidth can be measured in 0.9 ms. Test spectra of well-known polymers (polycarbonate and polystyrene) were recorded and compared to those of reference measurements with a standard FTIR spectrometer, confirming satisfying quality of the obtained spectra. Additionally, the spectral coding approach was compared to line-scans that were performed using single-bar illumination masks in order to demonstrate the multiplex advantage. The spectral noise could be reduced by a factor of 8.2 by using the spectral coding approach. The approach presented in this paper could potentially be used in process analytical applications and high-speed applications in general. These could be, for example, screening applications where specific spectral features are monitored with an adapted SROI and, once identified, a full spectrum can be taken for verification; target industries could thus be classical ones (chemical industry, pharmaceuticals, etc.), but also food and recycling industries. Besides the advantages in flexibility and spectroscopic performance, the proposed concept presents an interesting and effective alternative to expensive detector arrays. This aspect is even more relevant at higher wavelengths, such as the mid-infrared spectral region, where sensitive detector arrays are particularly expensive. Therefore, an extension of the presented near-infrared system into the mid-infrared, with the purpose of gas-sensing (CO_2_), is the subject of our future research.

## Data Availability

The data presented in this study are available on request from the corresponding author. The data are not publicly available due to legal reasons.
